# Activation of the central serotonergic system in response to delayed but not omitted rewards

**DOI:** 10.1111/j.1460-9568.2010.07480.x

**Published:** 2011-01

**Authors:** Kayoko W Miyazaki, Katsuhiko Miyazaki, Kenji Doya

**Affiliations:** 1 Neural Computation Unit, Okinawa Institute of Science and Technology1919-1 Tancha, Onna, Okinawa 904-0412, Japan; 2 Computational Neuroscience Laboratories, Advanced Telecommunications Research Institute InternationalKyoto, Japan

**Keywords:** dorsal raphe nucleus, microdialysis, rat, serotonin, waiting behaviour

## Abstract

The forebrain serotonergic system is a crucial component in the control of impulsive behaviours. However, there is no direct evidence for natural serotonin activity during behaviours for delayed rewards as opposed to immediate rewards. Herein we show that serotonin efflux is enhanced while rats perform a task that requires waiting for a delayed reward. We simultaneously measured the levels of serotonin and dopamine in the dorsal raphe nucleus using *in vivo* microdialysis. Rats performed a sequential food–water navigation task under three reward conditions: immediate, delayed and intermittent. During the delayed reward condition, in which the rat had to wait for up to 4 s at the reward sites, the level of serotonin was significantly higher than that during the immediate reward condition, whereas the level of dopamine did not change significantly. By contrast, during the intermittent reward condition, in which food was given on only about one-third of the site visits, the level of dopamine was lower than that during the immediate reward condition, whereas the level of serotonin did not change significantly. Dopamine efflux, but not serotonin efflux, was positively correlated with reward consumption during the task. There was no reciprocal relationship between serotonin and dopamine. This is the first direct evidence that activation of the serotonergic system occurs specifically in relation to waiting for a delayed reward.

## Introduction

Serotonin [5-hydroxytryptamine (5-HT)] has been implicated in impulsive behaviours (Evenden, 1999; Cardinal *et al.*, 2004). Forebrain serotonin depletion leads to more frequent choices of small, immediate rewards compared with large, delayed rewards (Wogar *et al.*, 1993; Bizot *et al.*, 1999; Mobini *et al.*, 2000; Denk *et al.*, 2005), a behavioural characteristic known as impulsive choice. However, there are apparently contradictory reports of only transient (Bizot *et al.*, 1999) or no (Winstanley *et al.*, 2003) effects on impulsive choice following 5-HT depletion. The existence of multiple serotonergic receptor systems and dynamic compensation mechanisms creates difficulties for lesion and pharmacological studies (Barnes & Sharp, 1999).

Based on a review of experimental data and theoretical models, we previously proposed that 5-HT controls the time scale of reward prediction, with the higher activity of 5-HT promoting consideration of further delayed rewards in action choice (Doya, 2002). Further, in a human functional magnetic resonance imaging study, we showed that the dorsal raphe nucleus (DRN) was activated when subjects learned to obtain large future rewards (Tanaka *et al.*, 2004). Manipulation of central serotonergic levels by dietary tryptophan depletion and loading showed that low serotonin levels steepen delayed reward discounting in humans (Schweighofer *et al.*, 2008). These results support our serotonin hypothesis. However, there has been little direct evidence showing that 5-HT neural firing or efflux is enhanced during behaviours in expectation of delayed reward as opposed to immediate reward (Winstanley *et al.*, 2006).

To elucidate the role of the ascending serotonergic system in natural goal-directed behaviours, we measured 5-HT and dopamine (DA) levels in the DRN of rats using *in vivo* microdialysis while the rat performed a sequential food–water navigation task in three different reward conditions: (i) the immediate reward condition in which rewards were delivered immediately after the rats entered the food and water sites; (ii) the delayed reward condition in which the rats had to wait up to 4 s at the reward sites before reward delivery; and (iii) the intermittent reward condition in which the rewards were delivered on only about one-third of the site visits. The third condition was introduced to test an alternative theory that serotonin is the opponent of DA, and thus encodes punishment or loss of reward (Deakin & Graeff, 1991; Daw *et al.*, 2002; Cools *et al.*, 2008).

There have been reports showing that release of 5-HT in the forebrain is not entirely uniform (Amat *et al.*, 1998; Maswood *et al.*, 1998; Winstanley *et al.*, 2006). We chose the DRN as the site for monitoring 5-HT efflux because the 5-HT release from the local collaterals within the DRN (Jacobs & Azmitia, 1992) should be proportional to the average firing rate of the 5-HT neurons that project from the DRN to the forebrain (Tao *et al.*, 2000). There is a practical advantage that the 5-HT concentration within the DRN is higher than in the target areas, such as the striatum and frontal cortex, so that we can perform reliable measurement without pre-treatment by selective serotonin reuptake inhibitor, which is often used in microdialysis measurement from the target areas (Winstanley *et al.*, 2006). In support of a positive relationship between the DRN 5-HT neuron firing and 5-HT release, during inescapable stress experiments, it has been reported that the c-fos was activated in DRN 5-HT neurons (Takase *et al.*, 2004) and that 5-HT efflux in the DRN was increased (Amat *et al.*, 2005). Furthermore, the DRN receives dopaminergic projection from the ventral tegmental area (Lee & Geyer, 1984; Kalen *et al.*, 1988). Thus, the DRN would be a suitable area for simultaneously examining the activity of 5-HT and DA neurons, although it is not clear how the release within the DRN is representative of the average firing of the ventral tegmental area DA neurons. We manipulated the timing and probability of reward delivery and examined: (i) how 5-HT and DA efflux in the DRN change during task performance, and (ii) what behavioural measures correlate with 5-HT and DA efflux.

## Materials and methods

### Subjects

All experimental procedures were performed in accordance with guidelines determined by the Okinawa Institute of Science and Technology (OIST) Experimental Animal Committee. Eleven male Long-Evans rats (Japan SLC, Hamamatsu, Japan), weighing 300–340 g at the start of the behavioural training time, were used in the present study. Animals were housed one per cage at 24 °C on a 12 : 12 h light:dark cycle (lights on 08:00 – 20:00 h). All training and test sessions were conducted during the light period, 5–6 days per week. The rats were deprived of food and water in the home cage, and received their daily food and water rations only during the experimental session (approximately 15 g/day and 20 mL/day, respectively).

### Behavioural apparatus and training

**Fig. 1:**
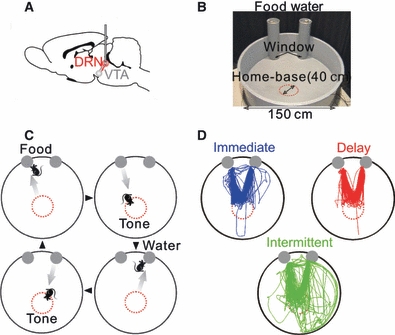
Experimental apparatus and design of the sequential food–water navigation task. (A) The position of the microdialysis probe. (B) Open field and reward cylinders (food site, water site) for the task. Windows for nose-pokes (reward locations) are indicated. The dotted line represents the unmarked home-base circle that the rat was required to enter prior to reward site visits. (C) Schematic of each rat's movements required to receive rewards at both sites. (D) Actual, typical trajectories of a rat during the immediate, delayed and intermittent reward conditions. VTA, ventral tegmental area.

We used a free operant task that we termed a sequential food–water navigation task. Six rats were individually trained and tested in a cylindrical apparatus (1.5 m in diameter with a 45-cm-high wall); two cylinders, denoted food site and water site, were fixed at a relative angle of 45° (Fig. 1B). When the rat poked its nose through a small window, a control photo beam was interrupted, and a small food pellet (45 mg) was delivered at the food site or a spout for water was protruded at the water site. The open field apparatus was surrounded by a sound-proof box (2.5 × 2.5 m and 2.4 m in height). Four 100 W lamps were set at four corners of the box. Four lamps on and two lamps on indicated the start and end of the task, respectively. One speaker was positioned above the open field. The position of the rat was monitored by a video tracking system (CV-2000; Keyence, Osaka, Japan). To receive a reward at both sites, the rat was required to traverse through an imaginary circle (home-base circle; φ = 40 cm) monitored by the tracking system between the food and water sites. An 8 kHz tone (tone 1 – 0.3 s, 70 dB) was presented when the rat entered the circle for 0.2 s, signalling that a reward was available at one of the cylinders. If the rat nose-poked at an incorrect site after tone 1, a 500 Hz tone (tone 2 – 0.3 s, 72 dB) was presented, signalling an error. Rats could start the next trial at any time after reward consumption. Rats were trained once per day for 2 h. It took 3 weeks or less for the rats to learn the sequential food–water navigation task.

### Surgery and cannulation

After rats had mastered the sequential food–water navigation task, they were anaesthetized with equithesin (3 mL/kg, i.p.) and stereotaxically implanted with a guide cannula (AG-8; Eicom, Kyoto, Japan) into the DRN (from bregma – posterior, −7.8 mm; lateral, 0 mm; ventral, −5.5 mm) according to the atlas of Paxinos & Watson (1998). The guide cannula was fixed onto the skull and anchored with dental acrylic and stainless steel screws. A dummy cannula (AD-8; Eicom) was inserted into the guide cannula and secured to the guide cannula with a cap-nut (AC-1; Eicom) to prevent infection and occlusions. Animals were housed individually after surgery and were allowed more than 1 week to recover.

### Task sequence with three reward conditions

After recovery from surgery, rats were retrained on a task sequence (30 min task performance followed by a 30 min rest period), consecutively, four or five times per day. After rats had mastered the task sequence, we introduced three reward conditions: immediate reward, delayed reward and intermittent reward. In the immediate reward condition, a rat could obtain a reward immediately after it nose-poked each rewarding site. In the delayed reward condition, rats had to keep their nose in the hold for 1 s in the first 2.5 min, 2 s in the next 2.5 min, and 4 s in the remaining 25 min before reward was delivered. Pulling out the nose for more than 500 ms before the end of this delay period caused a wait error, after which no reward was presented. In the intermittent reward condition, rats could obtain a food reward at approximately every third visit to the food site. In five out of 10 experiments a reward was given in one out of every three site visits, whereas in the other five experiments a reward was given quasi-stochastically. There was no significant difference in the behaviours of the two groups (time between tone presentation and food site nose-poke; Mann–Whitney *U*-test, *P*=0.17). Each reward condition lasted for 30 min, followed by a rest period for a further 30 min. Using these three reward conditions, two task sequences were prepared: task sequence 1, Immediate Reward (30 min) – rest (30 min) – Delayed Reward (30 min) – rest (30 min) – Intermittent Reward (30 min) – rest (30 min); and task sequence 2, Immediate Reward (30 min) – rest (30 min) – Intermittent Reward (30 min) – rest (30 min) – Delayed Reward (30 min) – rest (30 min). Subjects were divided into two groups. Before microdialysis measurements, each group experienced only one of two possible task sequences as a pre-test training, for five successive days. Each group experienced the same task sequence twice per day.

### Task sequence with reverse order and equated reward amount

In order to test any compound effects of the order of measurement and the amount of acquired reward on 5-HT efflux, we performed experiments with another task sequence in which the delayed reward condition was tested before the immediate reward condition and the number of trials during the 30 min periods was equated in the two conditions (task sequence 3). Five food-deprived rats were trained on the discrete trial task. In this task, one food site cylinder was placed in the open field (Supporting Information Fig. S1). During the 30 min task period, trials started every 30 s and rats could acquire 60 food pellets at a maximum. Four lamps on indicated the trial start. To receive a food pellet, the rat first had to enter the home-base circle for 0.2 s to present tone 1 before approaching the food site. When the rat nose-poked at the food site, two of four lamps were turned off until the next trial started. If the rat could not nose-poke to the food site within 20 s from the trial start, two of the four lamps also turned off until the next trial. Rats were trained by task sequence 3 [rest (30 min) – Delayed Reward (30 min) – rest (45 min) – Immediate Reward (30 min) – rest (30 min)] until rats could obtain more than seven food pellets every 5 min during the 30 min task. During training, task sequence 3 was repeated twice or three times and water was given for 5 min after task sequence 3 was finished. After the training, only water was given freely in the home cage. In the test session, rats executed task sequence 3 once or twice on each experimental day. After microdialysis probe implantation and stable baseline levels of 5-HT and DA were obtained, microdialysis measurement was executed by the following task sequence 3: rest for baseline measurement (30 min) – Delayed Reward (30 min) – rest (45 min) – Immediate Reward (30 min) – rest (30 min). Three rats executed task sequence 3 once on each day and two rats executed it twice on each day with more than a 90 min interval between the immediate reward condition in the first session and the delayed reward condition in the second session. After the microdialysis measurements had been completed (day 1), rats were placed into a container in the open field overnight and water was given freely. On experimental day 2, rats were tested with the same microdialysis probe. One of two rats that executed two task sequences per day was tested for 3 days (only one task sequence on day 3) using the same microdialysis probe and the other rat was tested for 1 day. In total, we obtained data from 13 task sessions over 10 days from five rats.

### In vivo microdialysis procedure

On the day of microdialysis measurements, a dialysis probe (A-I-8-02, length 2 mm, outer diameter 0.22 mm, 50 000 molecular-weight cut-off; Eicom) was calibrated with standard 5-HT and DA solution (20 mm NaH_2_PO_4_, 28 nm 5-HT and 33 nm DA) before each experiment. The probe recoveries (mean ± SEM) for 5-HT and DA were 9.5 ± 1.2% and 7.6 ± 1.0%, respectively (*n* = 6 for 5-HT and DA). The detection limit was about 30 fg per sample. After the calibration, the probe was carefully inserted into the guide cannula. The probe was secured to the guide cannula with a screw. The inlet and outlet of the probe were connected to a swivel (TCS2-23; Tsumura, Tokyo, Japan) through a free-moving tube (WT-20T; Eicom). Rats were placed in the open field until a stable baseline level of 5-HT and DA was obtained for at least 4 h. The probe was perfused at a constant flow rate of 2 μL/min with Ringer’s solution (147.2 mm NaCl, 4.0 mm KCl and 2.2 mm CaCl_2_; Wako, Osaka, Japan). The outflow was collected in a sample loop and injected automatically by an autoinjector (ESA-20; Eicom), once every 5 min, into a high-performance liquid chromatography apparatus with electrochemical detection (HTEC-500; Eicom). Extracellular 5-HT and DA levels in the DRN were simultaneously measured by the high-performance liquid chromatography with electrochemical detection every 5 min. 5-HT and DA were separated using an Eicompack PP-ODS column (4.6 mm i.d. × 30 mm; Eicom). The mobile phase contained 100 mm sodium phosphate buffer (pH 6.0; Wako), 2.0 mm sodium 1-decanesulphonate, 0.1 mm disodium EDTA (Dojindo, Kumamoto, Japan) and 1% (v/v) methanol (Wako). The flow rate was 500 μL/min and the system temperature was 25 °C. The concentrations of 5-HT and DA were measured by setting the working electrode at +400 mV against an Ag/AgCl reference electrode. Each probe was calibrated with standard 5-HT and DA solutions before each experiment. After stable baseline levels of 5-HT and DA were obtained, six baseline samples were collected. In the test session, rats performed the same task sequence that was experienced during pre-training. After the microdialysis measurements had been completed (day 1), rats were placed in the open field overnight. The perfusion rate of the modified Ringer’s solution was changed to 0.2 μL/min. At approximately 23 h after the previous day’s experiment had started, the flow rate of the Ringer’s solution was increased to 2 μL/min; it took approximately 60 min to stabilize 5-HT and DA baseline levels. On experimental day 2, rats were tested with the task sequence not used on day 1.

### Histology

Rats were deeply anaesthetized with 100 mg/kg sodium pentobarbital i.p. and then perfused with 0.9% NaCl followed by 10% formalin. The brains were removed and stored in 10% formalin for at least 24 h before being prepared as 60 μm coronal sections. Cresyl violet staining was used to help verify the placement of probe tracts.

### Data analysis

Neurochemical data were transformed into percent changes from baseline. For task sequences 1 and 2 during the food–water navigation task, the preparatory 30 min period of the immediate reward condition was used as the same baseline for each of the three reward conditions. For task sequence 3 during the discrete trial task, the preparatory 30 min period of the delayed reward condition was used as the same baseline for delayed and immediate reward conditions. Neurochemical data were analysed using a one-way repeated-measures anova followed by Tukey’s HSD test for multiple comparison. Statistical analyses were performed using statview or matlab statistical packages.

## Results

### Behaviours during three reward conditions

We monitored 5-HT and DA efflux in the DRN while rats performed the sequential food–water navigation task in an open field (1.5 m in diameter) (Fig. 1A and B). This task was free operant, and rats had to alternately visit two sites at which rewards could be obtained (food site and water site, centre angle 45°) via a home-base circle (40 cm in diameter) where a tone (8 kHz) was presented upon entry (Fig. 1C). Rats performed the task under three reward conditions: immediate reward, delayed reward and intermittent reward. In the immediate reward condition, rats could obtain a reward immediately after they nose-poked each rewarding site. In the delayed reward condition, rats had to wait up to 4 s between nose-poking and reward delivery. In the intermittent reward condition, a food reward was only available on approximately one-third of the visits to the food site. The characteristic trajectories that were observed during three reward conditions can be seen in Fig. 1D. Each reward condition lasted for 30 min, with 30 min rest periods between different conditions.

**Fig. 2:**
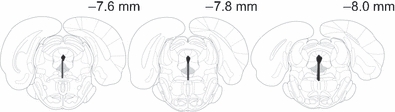
Locations of the microdialysis probes in the DRN. Black bars represent the 2 mm length of the dialysis membranes. Numbers beside each plate correspond to mm from bregma. Coronal drawing modified from Paxinos & Watson (1998).

**Fig. 3:**
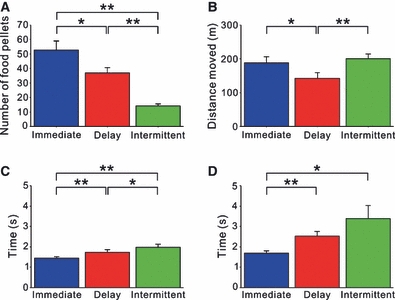
Behavioural results of the sequential food–water navigation task. (A) Mean numbers of food pellets acquired during each reward condition. (B) Mean distance traveled during the three reward conditions. (C) Time between tone presentation and food site nose-poke. (D) Time between tone presentation and water site nose-poke. All error bars show + SEM (*n*=10). Asterisks indicate significant differences, as assessed by the paired *t*-test, *P<0.05, **P<0.01.

Among the six rats, three of them were tested with sequence 1 on the first day and sequence 2 on the following day. Three others were tested in the counterbalanced order. We excluded the measurements in which the 5-HT or DA concentration data were smaller than signal-to-noise ratio 3. From this procedure, we obtained 10 task sequence data for 5-HT (5 from sequence 1 and 5 from sequence 2) and 8 task sequence data for DA (4 from sequence 1 and 4 from sequence 2). Probe placements within the DRN are shown in Fig. 2. The numbers of food pellets (mean ± SEM) obtained during the 30 min periods were 56.5 ± 6.5 (*n*=10) in the immediate reward condition, 37.7 ± 4.5 (*n*=10) in the delayed reward condition, and 14.8 ± 1.4 (*n*=10) in the intermittent reward condition (Fig. 3A). These differences are due, at least in part, to the time spent waiting in the delayed reward condition and the omission of the rewards in the intermittent reward condition. The total traveling distance during the 30 min period was significantly shorter in the delayed reward condition than in the other two conditions (paired *t*-test, immediate vs. delay *P*=0.011; immediate vs. intermittent, *P*=0.24; delay vs. intermittent, *P*=0.0011) (Fig. 3B), again partly due to the time spent waiting at the reward sites. Movement toward the reward sites was quickest during the immediate reward condition and slowest in the intermittent reward condition (paired *t*-test, *P*=0.0015 for time to food site; *P*=0.027 for time to water site) (Fig. 3C and D), suggesting the highest motivation in the immediate reward condition and the lowest motivation in the intermittent reward condition.

### 5-Hydroxytryptamine and dopamine efflux during task performance

**Fig. 4:**
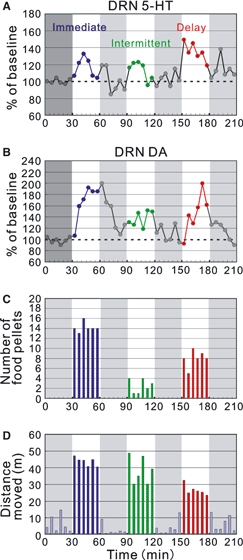
Changes in 5-HT and DA efflux in the DRN during a task sequence. A typical example of 5-HT (A) and DA (B) efflux and behavioural measures during task sequence 2. (C) Number of food pellets acquired and (D) distance moved every 5 min during rest and task periods are plotted corresponding to 5-HT and DA efflux levels. Grey areas indicate the rest period, and dark grey areas are the rest periods used as the baseline.

The average baseline levels of 5-HT and DA (corrected for *in vitro* probe recovery) (mean ± SEM) in the DRN were 7.6 ± 2.0 pg/10 μL (*n*=10) and 2.3 ± 0.4 pg/10 μL (*n*=8), respectively. An example of the time courses of 5-HT and DA efflux sampled every 5 min, and the corresponding behavioural measures, can be seen in Fig. 4. Both 5-HT and DA levels increased during the three reward conditions compared with the rest period. The 5-HT efflux was markedly higher in the delayed reward condition than in the immediate and intermittent reward conditions (Fig. 4A). However, the DA level was markedly lower in the intermittent reward condition than in the other two conditions (Fig. 4B), resembling the temporal profile of the number of food pellets acquired (Fig. 4C).

**Fig. 5:**
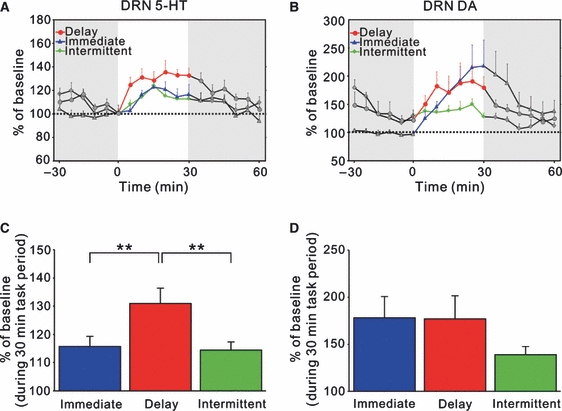
Average changes in 5-HT and DA efflux in the DRN during the three reward conditions. (A) Average time courses of 5-HT during the three reward conditions (*n*=10; ± SEM). The grey background shows the rest period. (B) Average time courses of DA during the three reward conditions (*n*=8; + SEM). (C) Average 5-HT levels during the 30 min task periods (*n*=10; + SEM). (D) Average DA levels during the 30 min task periods (*n*=8; + SEM). Asterisks indicate significant differences, as assessed by the Tukey’s HSD test, **P<0.01.

The average responses of 5-HT (*n*=10) and DA (*n*=8) can be seen in Fig. 5. The 5-HT level increased markedly from the beginning of the delayed reward condition compared with the other two conditions (one-way anova, *F*_2,158_ = 27.79, *P*<0.0001) (Fig. 5A). The DA level built up gradually during the immediate and delayed reward conditions, but was significantly less in the intermittent reward condition (one-way anova, *F*_2,126_ = 9.89, *P*=0.0001) (Fig. 5B). We also show the average time courses of 5-HT and DA efflux separately for task sequences 1 and 2 in Supporting Information Fig. S2. The average 5-HT and DA levels during the 30 min task periods relative to the baseline concentrations can be seen in Fig. 5C and D, respectively. The 5-HT levels were significantly elevated in all reward conditions compared with the baseline concentrations (16 ± 3.5% in the immediate reward condition, 31 ± 5.4% in the delayed reward condition, and 14 ± 2.9% in the intermittent reward condition; mean ± SEM, one-way anova, *F*_3,27_ = 25.11, *P*<0.0001 followed by Tukey’s HSD test, *P*<0.05). The 5-HT level in the delayed reward condition was significantly higher than that in the other two conditions (one-way anova, *F*_2,18_ = 19.00, *P*<0.0001 followed by Tukey’s HSD test, *P*<0.05). The DA levels were significantly elevated in immediate and delayed conditions compared with the baseline concentrations (one-way anova, *F*_3,21_ = 8.65, *P*=0.0006 followed by Tukey’s HSD test, *P*<0.05). For individual rats, five out of six rats showed significantly increased 5-HT efflux during the delayed reward condition compared with during the immediate and intermittent reward conditions (one-way anova, *P*<0.05 followed by Tukey’s test, *P*<0.05).

There was a significant difference in the average DA efflux among three reward conditions (one-way anova, *F*_2,14_ = 4.23, *P*=0.037). The average DA efflux was lower in the intermittent reward condition than in the immediate and delayed reward conditions, although it was not significant (Tukey’s HSD test, *P*>0.05).

In task sequences 1 and 2, the immediate reward condition always came first and there is a possibility that the 5-HT release increase during the delayed reward condition was simply due to surprise. Furthermore, as both task sequences 1 and 2 were free operant tasks, the number of acquired rewards was different among the three reward conditions, which may affect 5-HT efflux. In order to test these possibilities, we introduced task sequence 3 [rest (30 min) – Delayed Reward (30 min) – rest (45 min) – Immediate Reward (30 min) – rest (30 min)] in which the delayed condition was followed by the immediate condition and each trial was started every 30 s so that the number of trials were the same in the three conditions.

We obtained a total of 13 data sets of task sequence 3 in 10 days from five rats (two from four rats and five from one rat). We analysed the nine data sets from the first session on experimental days 1 and 2 (two from four rats and one from one rat). Rats obtained more than nine food pellets every 5 min during the delayed and immediate reward conditions, and there was no significant difference in the number of acquired food pellets (one-way anova, *F*_11,88_ = 0.77, *P*=0.67) (Supporting Information Fig. S3). Supporting Information Fig. S4A shows the average time course of 5-HT efflux during task sequence 3. The average 5-HT levels during the 30 min task periods increased in both reward conditions compared with baseline (37 ± 6.4% in the delayed reward condition, 24 ± 4.2% in the immediate reward condition; mean ± SEM, one-way anova, *F*_2,16_ = 24.4, *P*<0.0001 followed by Tukey’s HSD test, *P*<0.05) and the increase in 5-HT efflux during the delayed reward condition was larger than during the immediate reward condition (paired *t*-test, *P*=0.026) (Supporting Information Fig. S4B). The average 5-HT level during the delayed reward condition was also significantly larger than during the immediate reward condition for data sets of the first task sequence 3 of experimental day 1 (five data sets from five rats; paired *t*-test, *P*=0.046) and all 13 data sets obtained (paired *t*-test, *P*=0.0097). This result rejects the possibility that the increased 5-HT efflux in the delayed reward condition in sequences 1 and 2 was due to the order effect or the difference in the amount of acquired reward.

The 5-HT efflux during task sequence 3 was slightly higher than that during task sequences 1 and 2 in both conditions (37 vs. 31% from the baseline during the delayed reward condition, 24 vs. 16% during the immediate reward condition). One possible reason is that the rats additionally had to wait for the trial start tone at around the home-base circle for both the immediate and delayed conditions, which might contribute to extended 5-HT activity in both conditions.

### Correlation between 5-hydroxytryptamine and dopamine

**Fig. 6:**
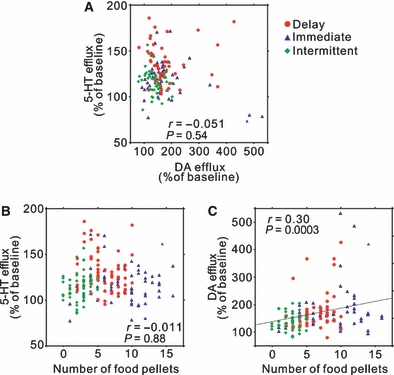
Correlations between 5-HT and DA efflux and the number of food pellets. (A) Comparison between 5-HT and DA efflux. (B) Comparison between 5-HT efflux and the number of food pellets acquired in each 5 min sample period. (C) Comparison between DA efflux and the number of food pellets acquired in 5 min.

To test whether there was any reciprocal interaction between 5-HT and DA, as predicted from the opponent theory, we examined the correlation between 5-HT and DA efflux every 5 min. There was no significant negative or positive correlation between 5-HT and DA efflux (*r*= −0.051, *P*=0.54) in any of the three reward conditions or in any of the conditions combined (Fig. 6A). We further checked whether the 5-HT and DA efflux correlated with the behavioural measures of food consumption and distance moved every 5 min. Although there was no significant correlation between 5-HT efflux and food consumption (*r*= −0.011, *P*=0.88) (Fig. 6B), DA efflux was significantly correlated with food consumption (*r*=0.30, *P*=0.0003) (Fig. 6C). 5-HT efflux had a significant negative correlation with distance moved during the task (*r*= −0.24, *P*=0.0011). However, a low level of 5-HT efflux would not be simply related to locomotion, as 5-HT efflux was lowest during the rest period (Fig. 4A and D). DA efflux had no significant correlation with distance moved (*r*= −0.14, *P*=0.09). Neither 5-HT nor DA efflux was significantly correlated with the time between the tone presentation at the home-base circle and the nose-poke at the food site (*r*=0.12, *P*=0.12 and *r*= −0.11, *P*=0.19, respectively).

## Discussion

We monitored the 5-HT and DA efflux in the DRN of rats performing the food–water navigation task in which we independently manipulated the reward conditions. The principal observation of the present study was that the level of 5-HT significantly increased when rats worked in expectation of delayed rewards, but not when rewards were omitted. This is the first direct evidence of activation of the forebrain serotonergic system specifically in relation to waiting for a delayed reward, which is consistent with the theory that serotonin regulates the time scale of future reward prediction (Doya, 2002).

The alternative theory assumes that 5-HT is the opponent of DA (Deakin & Graeff, 1991; Daw *et al.*, 2002; Cools *et al.*, 2008). As it is well known that DA reports predicted reward (Schultz, 1998, 2007; Satoh *et al.*, 2003; Nakahara *et al.*, 2004), the theory posits that 5-HT should report predicted punishment or loss of reward (Daw *et al.*, 2002). Although this explains the link between a high level of 5-HT and anxiety, it is at odds with the anti-depressant effects of 5-HT-enhancing drugs, and cannot explain a more recently shown link between lower 5-HT and impulsive behaviours (Evenden, 1999; Cardinal *et al.*, 2004).

Our observation that the level of DA in the DRN was correlated with the amount of acquired reward reconfirms the previous findings of increased DA efflux in the medial prefrontal cortex and nucleus accumbens during reward consumption (Bassareo & Di Chiara, 1997; Ahn & Phillips, 1999). This observation is also consistent with neural recording studies showing that dopaminergic neurons fire upon delivery of unpredicted rewards or presentation of reward predictive cues (Schultz, 1998, 2007; Satoh *et al.*, 2003; Nakahara *et al.*, 2004), because the sum of the predicted and unpredicted components or rewards matches the total rewards delivered. According to the theory that the 5-HT is the opponent of DA (Deakin & Graeff, 1991; Daw *et al.*, 2002; Cools *et al.*, 2008), the 5-HT efflux should have an opposite response profile to that of DA, with an increase in the intermittent reward condition. In the present study, 5-HT efflux was significantly increased during the delayed reward condition, but not during the intermittent reward condition, when compared with the immediate reward condition. Furthermore, the lack of a negatively correlated interaction between 5-HT and DA contrasts with the opponent theory. These results are consistent with our theory stating that 5-HT and DA represent orthogonal information, 5-HT for the time scale of reward expectation and DA for the amplitude of reward expectation (Doya, 2002).

### Dorsal raphe nucleus 5-hydroxytryptamine neural activity and impulsivity

Impulsivity can be broadly divided into impulsive action and impulsive choice (Evenden, 1999). Impulsive action is the inability to inhibit undesired actions, whereas impulsive choice is the tendency to choose small, immediate rewards over large, delayed rewards. The role of the serotonergic system in impulsivity has been studied mainly using forebrain 5-HT depletion and pharmacological treatment with 5-HT receptors and transporters, with contradictory results reported. For instance, selective serotonin reuptake inhibitors resulted in increased selection of the large delayed reward (Bizot *et al.*, 1988, 1999), whereas a nonselective 5-HT receptor antagonist promoted self-controlled choice (Evenden & Ryan, 1996). Forebrain 5-HT depletion has been shown to lead to greater choice of small, immediate rewards compared with large, delayed rewards (Wogar *et al.*, 1993; Bizot *et al.*, 1999; Mobini *et al.*, 2000; Denk *et al.*, 2005), whereas systemic treatment with the 5-HT_1A_ receptor agonist 8-hydroxy-2-(di-n-propylamino) tetralin, which is known to suppress 5-HT neuron firing through autoreceptors, produced impulsive choice (Liu *et al.*, 2004; Winstanley *et al.*, 2005). However, a recent rodent study demonstrated that forebrain 5-HT depletion caused increased motor impulsivity, but not delay discounting (Harrison *et al.*, 1997; Winstanley *et al.*, 2004). Finally, opposing effects of 5-HT_2A_ and 5-HT_2C_ receptor subtypes have been reported on motor impulsivity (Fletcher *et al.*, 2007). A potential explanation for these contrasting data is the existence of multiple pre-synaptic and post-synaptic serotonergic receptor subtypes in the target areas of serotonergic projections, and also dynamic compensation mechanisms dependent on how 5-HT was manipulated (i.e. by depletion and/or pharmacological treatment).

There is only a little direct evidence of endogenous changes in 5-HT release in relation to impulsive or self-regulated behaviours. The present study provides direct evidence that serotonergic neurons in the DRN enhance their activity specifically when the animal is working for delayed rewards rather than immediate rewards. An increase in 5-HT release in the medial prefrontal cortex has been reported in correlation with impulsive actions in a visual attention task (Dalley *et al.*, 2002), although these data contrast with lesion studies showing that 5-HT depletion increases premature responses in the same task and the five-choice serial reaction time task (Harrison *et al.*, 1997; Winstanley *et al.*, 2004). A recent study reported a significant increase in 5-HT efflux in the medial prefrontal cortex of rats performing a delay-discounting task when compared with ‘yoked’ rats performing no choice (Winstanley *et al.*, 2006). However, that study was unable to show any correlation between 5-HT levels and delay lengths or animals’ choices. The same group reported that systemic 8-hydroxy-2-(di-n-propylamino) tetralin treatment promoted impulsive choice in this delay-discounting task (Liu *et al.*, 2004; Winstanley *et al.*, 2005), whereas forebrain 5-HT depletion did not (Winstanley *et al.*, 2003, 2004). A potential explanation for this discrepancy is that a functionally intact 5-HT system is specifically required for withholding competing actions in anticipation of a forthcoming reward (Harrison *et al.*, 1997; Winstanley *et al.*, 2004); a marked difference between our task and the delay-discounting task is that, in our task, the rats had to keep nose-poking in the hole until reward delivery, whereas in the delay-discounting task rats were free to do anything between making a choice and the reward delivery.

### Dorsal raphe nucleus 5-hydroxytryptamine neural activity and delayed reward evaluation

We previously reported increased neural activity in the medial prefrontal cortex during the delay period preceding forthcoming rewards (Miyazaki *et al.*, 2004). Human functional magnetic resonance imaging studies show differential involvement of brain areas in the prediction of immediate and delayed rewards, with the dorsal striatum and prefrontal cortex showing consistent activity for delayed rewards (McClure *et al.*, 2004; Tanaka *et al.*, 2004, 2006). A recent functional magnetic resonance imaging study using tryptophan depletion and loading showed that the dorsal striatal activity correlated with long-term reward prediction was enhanced with the activation of the serotonergic system (Tanaka *et al.*, 2007). In the present study, during successful performance in the delayed reward condition, rats chose to keep nose-poking against other possible behaviours, such as resting, exploring or grooming, in expectation of forthcoming food or water reward, which requires a long enough evaluation time window for future rewards. The results from the present study are in support of the hypothesis that increased activity of 5-HT neurons in the DRN signals a longer setting of the time scale of reward evaluation (Doya, 2002) and facilitates behaviours in expectation of delayed rewards through their dense projection to the striatum and prefrontal cortex. Local injection of 5-HT_1A_ receptor agonist to the DRN to reduce 5-HT neural activity without affecting 5-HT_1A_ receptors in the target areas might be an effective way to examine the role of 5-HT neural activity in working for delayed reward. Recording of 5-HT neural activity during a similar delayed reward task would clarify the role of 5-HT in specific task events.

Our finding of a link between serotonergic activation and behaviours in expectation of future reward has important medical implications, as it may explain the effectiveness of serotonin-enhancing drugs for impulsive behaviours (Grant & Potenza, 2004). The next important questions are what is the control principle and what is the network mechanism for regulating serotonergic activity in response to environmental conditions and internal motivation.

## Abbreviations

5-HT: 5-hydroxytryptamine
DA: dopamine
DRN: dorsal raphe nucleus

## Supporting Information

**Fig. S1.** Schematic of rats' movements in the experiment with the task sequence 3.

**Fig. S2.** Average time courses of 5-HT and DA efflux in the DRN during the task sequences 1 and 2 in the food-water navigation task.

**Fig. S3.** Average number of food pellets acquired every 5 min during the task sequence 3 (*n* = 9; ± SEM).

**Fig. S4.** 5-HT efflux in the DRN during the task sequence 3.

Please note: As a service to our authors and readers, this journal provides supporting information supplied by the authors. Such materials are peer-reviewed and may be re-organized for online delivery, but are not copy-edited or typeset by Wiley-Blackwell. Technical support issues arising from supporting information (other than missing files) should be addressed to the authors.
