# Supplementary material for: Activation of the central serotonergic system in response to delayed but not omitted rewards
Source: Eur J Neurosci. 2011 Jan;33(1):153–60. doi: 10.1111/j.1460-9568.2010.07480.x (PMC3040841; doi:10.1111/j.1460-9568.2010.07480.x)
Supplement: Supplementary file 1 [file ejn0033-0153-SD1.doc]

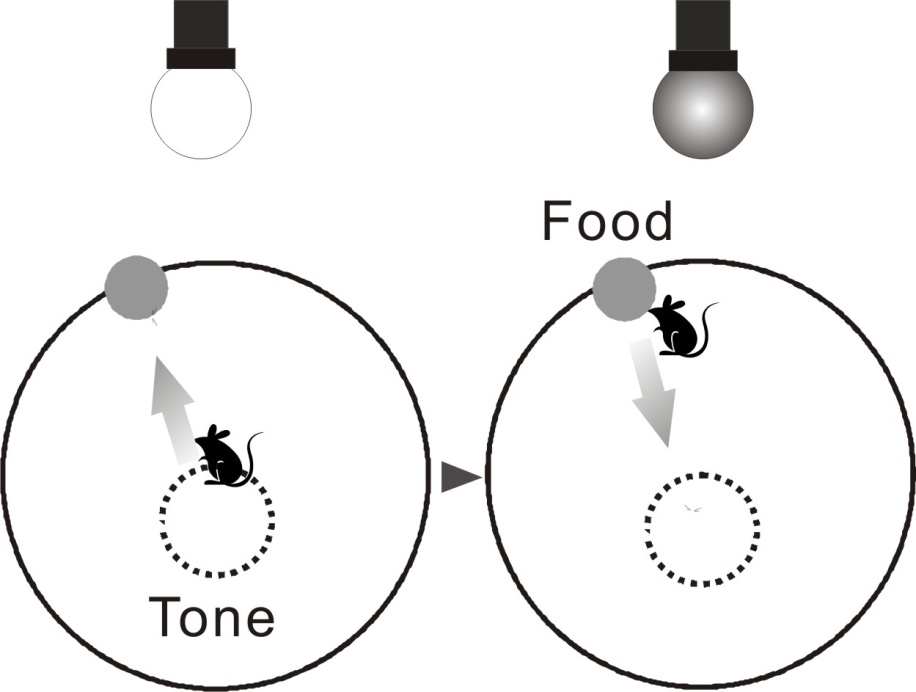


**Fig. S1.** Schematic of rats’ movements in the experiment with the task sequence 3. Each trial is started every 30 seconds when the light is lit. If the rat is in the home circle, a tone is presented. The light is turned off when the rat nosepokes to the food site, where a food pellet is given after a delay or immediately.
