# Supplementary material for: Activation of the central serotonergic system in response to delayed but not omitted rewards
Source: Eur J Neurosci. 2011 Jan;33(1):153–60. doi: 10.1111/j.1460-9568.2010.07480.x (PMC3040841; doi:10.1111/j.1460-9568.2010.07480.x)
Supplement: Supplementary file 2 [file ejn0033-0153-SD2.doc]

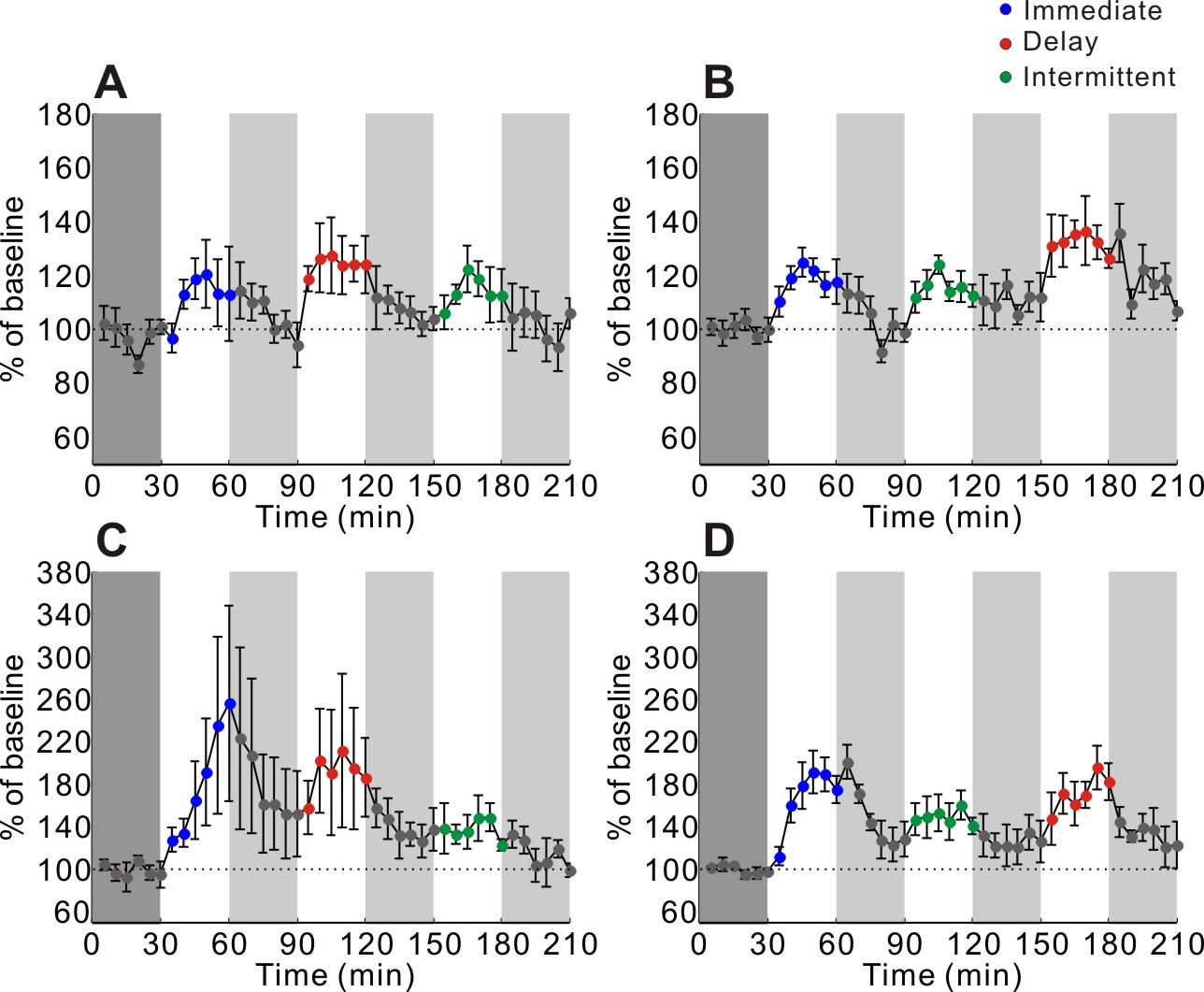


**Fig. S2.** Average time courses of 5-HT and DA efflux in the DRN during the task sequences 1 and 2 in the food-water navigation task. (A) 5-HT efflux during the task sequence 1 (*n* = 5; ±SEM). (B) 5-HT efflux during the task sequence 2 (*n* = 5; ± SEM). (C) DA efflux during the task sequence 1 (*n* = 4; ± SEM). (D) DA efflux during the task sequence 2 (*n* = 4; ± SEM). Gray areas indicate rest periods, and the dark gray areas are the rest periods used as the baseline.
