# Supplementary figures and images for: Activation of the central serotonergic system in response to delayed but not omitted rewards
Source: Eur J Neurosci. 2011 Jan;33(1):153–60. doi: 10.1111/j.1460-9568.2010.07480.x (PMC3040841; doi:10.1111/j.1460-9568.2010.07480.x)

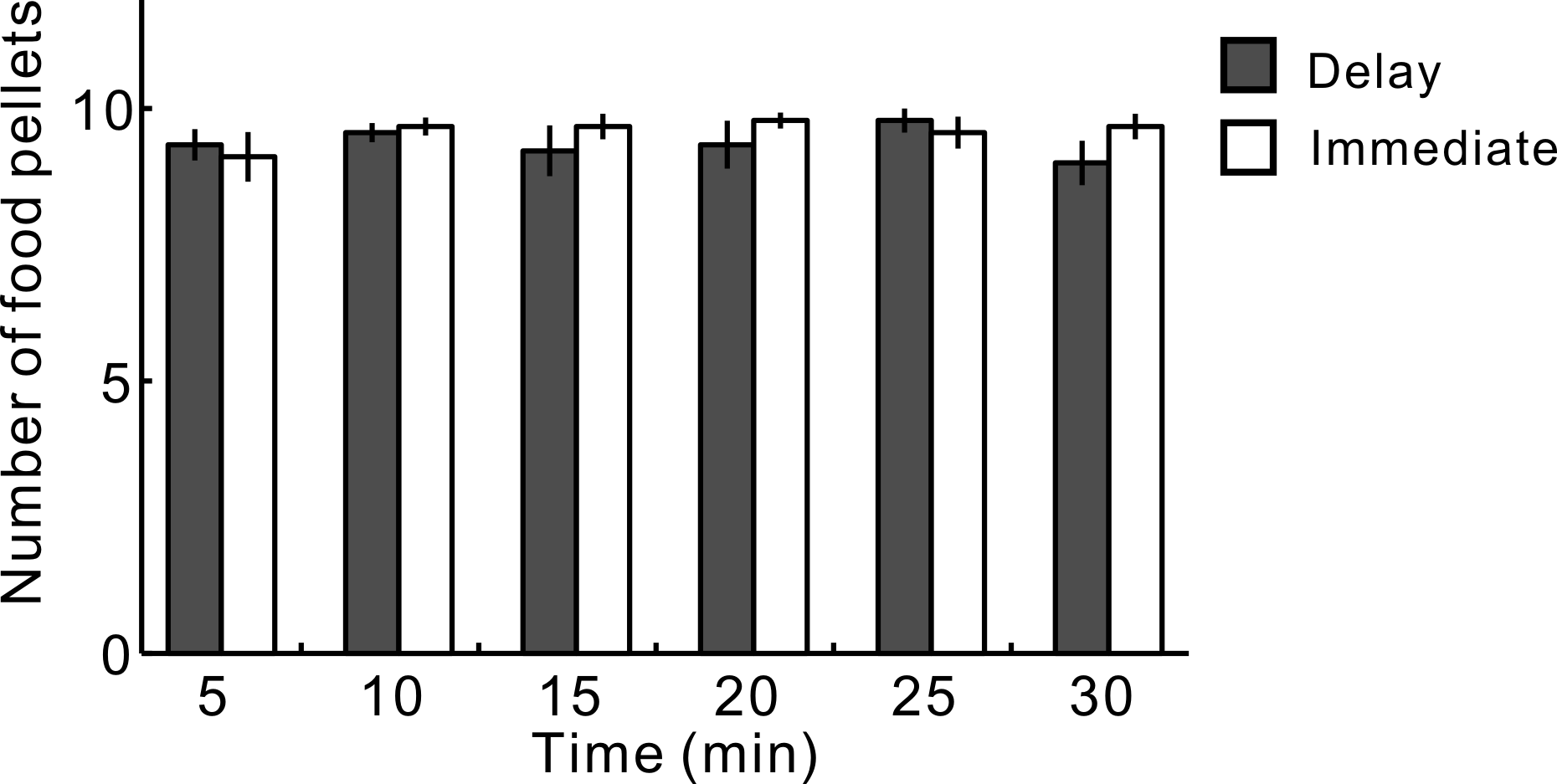


**Fig. S3.** Average number of food pellets acquired every five min during the task sequence 3 (*n* = 9; ± SEM).

Supplement: Supplementary file 3 [file ejn0033-0153-SD3.doc]
