# Supplementary material for: Activation of the central serotonergic system in response to delayed but not omitted rewards
Source: Eur J Neurosci. 2011 Jan;33(1):153–60. doi: 10.1111/j.1460-9568.2010.07480.x (PMC3040841; doi:10.1111/j.1460-9568.2010.07480.x)
Supplement: Supplementary file 4 [file ejn0033-0153-SD4.doc]

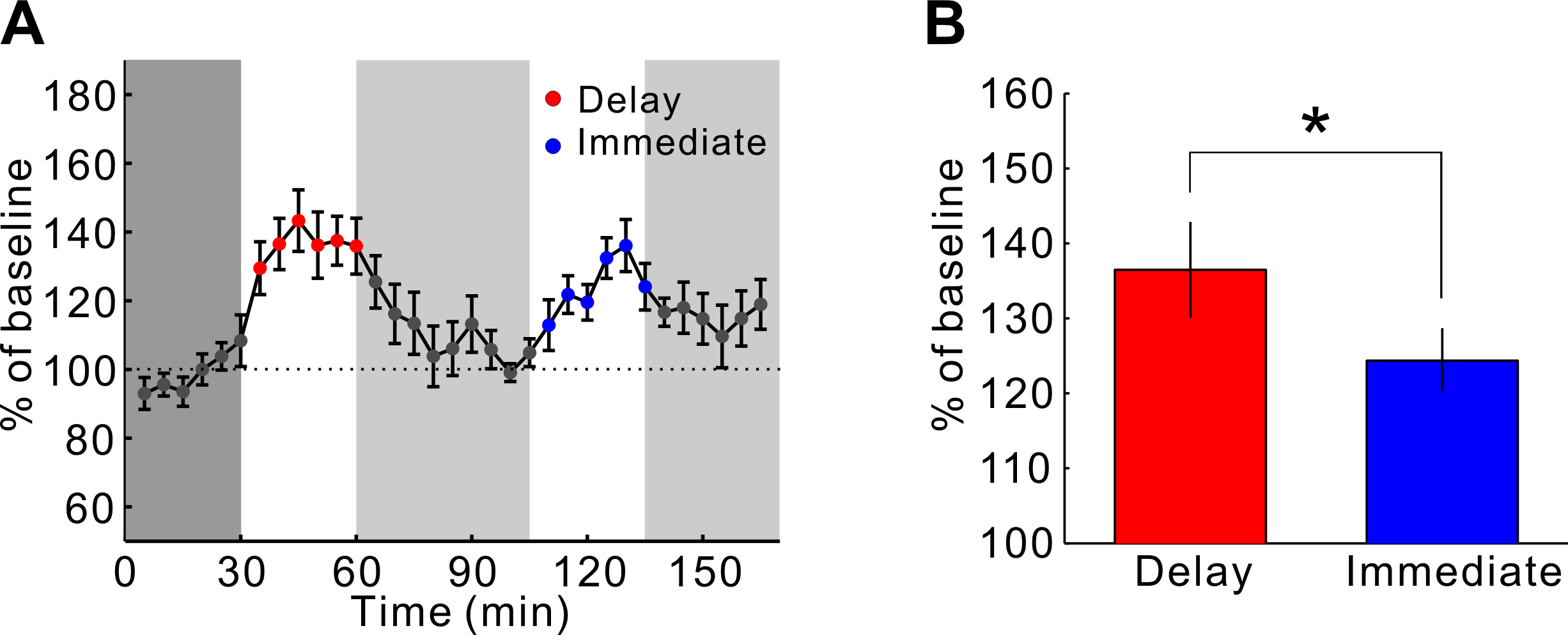


**Fig. S4.** 5-HT efflux in the DRN during the task sequence 3. (A) Average time course of 5-HT efflux during the task sequence 3 (*n* = 9; ± SEM). Gray areas indicate rest periods, and the dark gray area shows the rest period used as the baseline.(B) Average 5-HT levels during the 30 min task periods (*n* = 9; ± SEM). Asterisks indicate significant differences, as assessed by the paired t-test, **P* < 0.05.
